# Urbanization alters soil trace metal enrichment and health risks in the black soil Region of Northeast China

**DOI:** 10.1371/journal.pone.0346565

**Published:** 2026-04-06

**Authors:** Guanxin Du, Yu Sun, Hongyan Lu, Baiquan Yan, Huimin Dai, Kai Liu, Keke Xu

**Affiliations:** 1 Earth Science College, Northeast Petroleum University, Daqing, Heilongjiang, China; 2 National Key Laboratory of Continental Shale Oil, Northeast Petroleum University, Daqing, Heilongjiang, China; 3 China Key Laboratory of Black Soil Evolution and Ecological Effects, Ministry of Natural Resources, Shenyang, Liaoning, China; 4 Liaoning Provincial Natural Resources Affairs Service Center, Shenyang, Liaoning, China; 5 Shenyang Center of Geological Survey, China Geological Survey, Shenyang, Liaoning, China; Ardakan University, IRAN, ISLAMIC REPUBLIC OF

## Abstract

While urbanization is accelerating across China’s black soil regions, its specific impact on trace metal accumulation in the vulnerable urban-agricultural transition zones remains poorly understood. This study investigated such a zone in Arongqi, analyzing 32 topsoil samples (0–20 cm) from three subzones for concentrations of Cd, Cu, Pb, Zn, As, Hg, Ni, Cr, Sb, Ba, Co, Mo, Sr, V, Tl, Ag, pH, and soil organic matter (SOM). Ecological and health risks were evaluated using enrichment factors, the pollution load index (PLI), and health risk models. Pollution sources were apportioned via correlation analysis, principal component analysis, and positive matrix factorization. Results showed that urbanization did not exacerbate soil acidification but significantly reduced SOM, resulting in significant enrichment of Cd, Pb, Zn, Ba, Co, Mo, Sr, V, and Ag relative to background levels. The overall pollution status was moderate (PLI), yet distinct spatial patterns emerged: the Urbanizing Zone showed mild contamination by Cd, Ba, and Mo; the Peri-urban Arable Land by Cd, Mo, Sr, V, and Ag; and the Urban Zone by Cd, Pb, Ba, and Sr. The total carcinogenic risk was moderate for both children and adults, with higher risk for adults in the Peri-urban Arable Land and for children in the Urban Zone. Oral ingestion and dermal contact were the dominant exposure pathways, with Cr and As being the key carcinogenic factors for children and Cr also a significant risk for adults. Source apportionment identified five major contributors: traffic emissions (27.46%), soil parent material (24.77%), contemporary agricultural activities (24.54%), urban combustion (11.22%), and historical agricultural practices (11.22%). This study provides a new, spatially-resolved perspective for risk assessment and source management during the urbanization of black soil regions.

## 1. Introduction

Modern industrial and agricultural activities, along with rapid urban development, seriously threaten soil environmental safety and human health, becoming a global concern [1–3]. Harmful heavy metals in soil exhibit low mobility, high biotoxicity, and resistance to degradation [4]. Pollutants originating from agricultural activities, urban combustion, and transportation enter the human body through inhalation, ingestion, and dermal contact, accumulating over time in organs such as the brain, liver, and kidneys, posing health risks [5,6]. With the acceleration of urbanization in China’s black soil regions, the enrichment of heavy metals in the area has become increasingly evident [7]. Therefore, a deep understanding of the enrichment, source analysis, and risk assessment of pollutants in black soil under urban expansion is crucial for sustainable agricultural development strategies in Northeast China’s black soil regions.

Land use type is a primary factor controlling metal accumulation. Agricultural soils are often enriched with Cu, Zn, Pb, and Hg due to fertilizer and pesticide application [8], while urban soils tend to accumulate metals like Ni, Cu, Zn, and Co from industrial, domestic [9], and traffic emissions [10]. The urban-rural fringe, as a dynamic transition zone, presents a more complex and heterogeneous pollution profile than purely urban or agricultural landscapes [11]. Studying such gradients is critical for understanding heavy metal enrichment mechanisms during land-use change [12,13]. However, many existing studies focus on static land types, potentially overlooking the legacy pollution and evolving source contributions inherent to ongoing urbanization processes [14]. Consequently, systematic investigations that track pollution characteristics across an urbanization gradient—from rural farmland to urban core—remain scarce for black soil regions.

Accurately assessing contamination requires an integrated methodological framework. Ecological indices like the Enrichment Factor (EF) [15] and Pollution Load Index (PLI) [16] effectively quantify the degree of anthropogenic influence and the overall pollution level, respectively. To evaluate the direct impact on human health, health risk assessment models—which calculate lifetime carcinogenic risks and non-carcinogenic hazard quotients via specific exposure pathways such as ingestion, dermal contact, and inhalation—have become a cornerstone tool linking soil pollution to public health decision-making [17,18]. This established methodology has been widely applied to assess heavy metal exposure risks in soils under various land-use types, including urban, agricultural, and industrial areas [19,20]. Unlike ecological assessments that focus on environmental baselines, health risk evaluation directly quantifies potential hazards to sensitive population, providing indispensable evidence for risk-based soil management strategie [21,22]. Given the limitations of single-method evaluations, a combined application of ecological and health risk assessment methods allows for a more comprehensive and multi-dimensional interpretation of pollution status and its implications [23].

In source apportionment, receptor models are commonly used to quantify the contributions of different sources to the samples [24], such as principal component analysis (PCA) [25] and positive matrix factorization (PMF) [26]. Principal component analysis (PCA) reduces the dimensionality of data to form new components linked to the source data [27]. PMF maximizes the use of dataset information by applying bootstrapping to address sampling and analytical uncertainties, and is an official quantitative source apportionment receptor model recommended by the U. S. Environmental Protection Agency (US EPA) [28]. The combination of both has been widely applied in source apportionment for air [29], water [30], and soil [31]. The mixed model calculates the contribution rates of pollution sources, comparing the similarities and differences between different pollution source components to mutually verify and provide more reliable source contributions [32].

Despite the availability of these established methods, a holistic study that integrates spatial gradient analysis, multi-method risk assessment, and quantitative source apportionment is notably lacking for urbanizing black soil landscapes. This study aims to address this gap by examining a typical urban-agricultural transition zone in Arongqi within China’s black soil region. The specific objectives are to: (1) quantify the spatial distribution and enrichment levels of trace metals along an urbanization gradient; (2) evaluate the ecological and human health risks posed by these metals, differentiating between children and adults; and (3) assess and quantify the contributions of potential pollution sources along this gradient using receptor-based source apportionment. The findings are expected to provide a scientific basis for targeted soil management during land-use conversion in these ecologically sensitive black soil areas.

## 2. Materials and methods

### 2.1. Study area

**Fig 1:**
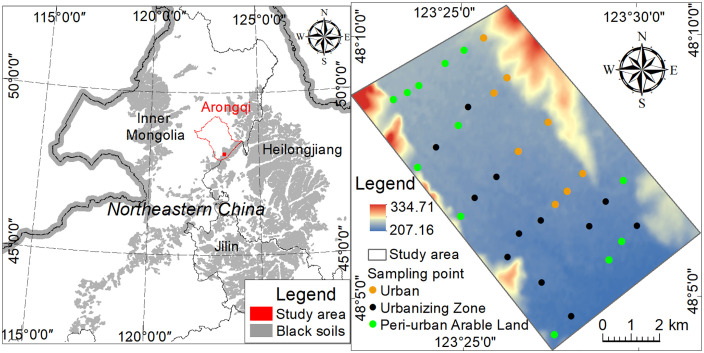
Map of study area (a) and sampling locations (b), The base map was adapted from Xu [33], which is licensed under a CC BY 4.0 license.

The research area is located in Arongqi City, Inner Mongolia Autonomous Region, China (Fig 1a).The study area has a semi-humid continental monsoon climate, with elevations between 260 and 350 m and terrain sloping downward from northeast to southwest. The mean annual temperature is 3.4°C, and annual precipitation ranges from 400 to 450 mm, mainly falling between June and August. Located in the northwest of China’s black soil region, the area is dominated by black soils and chernozems. Agriculture is a key economic sector, with a high proportion of arable land. However, rapid urbanization has converted large areas of farmland into construction land. Over the past four decades, an urban–agricultural transition zone has developed, comprising built-up urban areas, newly urbanizing zones, and peri-urban cropland—a pattern typical of the Northeast Black Soil Region [34]. Additionally, the surrounding area is rich in non-metallic minerals. Mining activity spanning more than 20 years has left numerous abandoned mines [35], which may increase the risk of toxic element contamination in soils due to land-use change. Residents in this transition zone may be directly exposed to contaminated soil through daily activities, home gardening, and dust from land disturbance.

### 2.2. Sample Collection and Analysis

#### 2.2.1. Sample Collection.

In April 2019, systematic field sampling was conducted within the delineated urban-agricultural transition zone of Arun Banner. A total of 32 composite topsoil samples (0–20 cm depth) were collected by digging pits from three functional zones: urban area (n = 8), urbanized area (n = 12), and suburban farmland (n = 12) (Fig 1b). Sampling sites were carefully designed to cover the spatial heterogeneity of each zone and to avoid proximity to roads, buildings, or other obvious point sources, thereby reflecting regional background conditions. At each sampling location, a composite sample was formed by thoroughly mixing five sub-samples collected from the four corners and the center of a 10 m × 10 m square. Approximately 1 kg of soil was then obtained by quartering. After removing plant roots, gravel, and other debris, the samples were air-dried naturally, sieved through a 20-mesh (<0.84 mm) nylon sieve, and subsequently homogenized with an agate mortar. The homogenized material was passed through a 200-mesh (<0.074 mm) nylon sieve before being sent to the laboratory. The sample testing and analysis were performed by the Northeast Mineral Resources Supervision and Testing Center of the Ministry of Natural Resources of China.

No specific permits were required for the observational fieldwork described here, as the study was conducted on publicly accessible trails and did not involve the collection of protected species or any disturbance to the environment.

#### 2.2.2. Physicochemical property and elemental analysis of samples.

Soil organic matter (SOM) content was analyzed using the Walkley–Black method [36]. Soil pH was measured potentiometrically with a calibrated pH meter (PHS-3C, INESA, China) using a soil-to-deionized water ratio of 1:2.5 (weight/volume). The suspension was equilibrated for 30 minutes before measurement.

Trace elements were digested using a mixture of HNO₃ (6 mL), HCl (2 mL), and HF (1 mL) in sealed polytetrafluoroethylene vessels via microwave-assisted acid digestion. The digestion program involved ramping to 180°C within 15 minutes and holding for 20 minutes. After cooling, the digest was transferred to a polytetrafluoroethylene beaker, heated to near dryness to expel HF, and the residue was reconstituted with 2% HNO₃ and diluted to 50 mL. Concentrations of 16 trace elements (Cd, Cu, Pb, Zn, As, Hg, Ni, Cr, Sb, Ba, Co, Mo, Sr, Ag, V, Tl) were determined using inductively coupled plasma mass spectrometry (ICP-MS, iCAP RQ, Thermo Fisher Scientific, USA). Method detection limits (MDLs), calculated as three times the standard deviation of procedural blanks, were (in mg/kg): 0.02 (Cd), 1 (Cu), 2 (Pb), 4 (Zn), 0.2 (As), 0.005 (Hg), 1 (Ni), 0.2 (Cr), 0.05 (Sb), 0.05 (Ba), 0.2 (Co), 0.2 (Mo), 2 (Sr), 0.02 (V), 0.1 (Ag), and 0.05 (Tl).

Stringent QA/QC protocols were implemented throughout the analytical process. For every batch of eight samples, one procedural blank, one duplicate sample, and one certified reference material (CRM: GBW07405 from the Institute of Geophysical and Geochemical Exploration, China) were processed simultaneously. The spike recoveries for all elements in the CRM ranged from 85% to 115%, with most falling between 90% and 110%, confirming the accuracy of the digestion and analytical procedures. The relative percentage difference (RPD) between duplicate samples was consistently below 10%, indicating good analytical precision. All reported elemental data were blank-corrected.

### 2.3. Data processing

Data organization and analysis were conducted using Microsoft Excel, Origin, and SPSS version 22, with correlation analysis (PCA) and significance analysis performed using R Studio software. The positive matrix factorization (PMF) source analysis was carried out using EPA PMF 5. 0. Figures were created using CorelDraw X9 and ArcMap.

### 2.4. Pollution assessment method

#### 2.4.1. Enrichment factor method.

The enrichment factor is commonly a reliable method in soil heavy metal assessment, with the following pollution level classification: no pollution: EF ≤ 1; light pollution: 1 < EF ≤ 2; moderate pollution: 2 < EF ≤ 5; severe pollution: 5 < EF ≤ 20; very severe pollution: 20 < EF ≤ 40. [15]. The formula is as follows (1):


$$EF=\frac{{\left(C_{i}/C_{n}\right)}_{a}}{{\left(C_{i}/C_{n}\right)}_{b}}$$ (1)

Where C_i_ is the measured concentration of heavy metal element i; C_n_ is the concentration of the reference element (Fe was selected as the reference element in this study); a and b represent the measured heavy metal concentration and the corresponding element’s standard value, respectively. In this study, the background values of soil elements in Inner Mongolia were used [37].

#### 2.4.2. Pollution load index.

The pollution load index method was proposed by Tomlinson et al. in 1980 as an analytical method for assessing heavy metal pollution levels. It has a stable effect on both point and regional-scale heavy metal evaluation. The pollution load index classification is as follows: No pollution: PLI < 1; Moderate pollution: 1 < PLI < 2; Strong pollution: 2 < PLI < 3; Very strong pollution: PLI ≥ 3 [16]. The formulas are as follows (2)(3)(4):


$${CF}_{i}=C_{i}/C_{oi}$$ (2)

$$PLI\;=\;\sqrt[{n}]{{CF}_{\text{1}}\;\times \;{CF}_{\text{2}}\;\times \;.\;.\;.\;{\times CF}_{n}}$$ (3)

$${PLI}_{area}=\sqrt[{m}]{{PLI}_{\text{1}}\;\times \;{PLI}_{\text{2}}\;\times \;.\;.\;.\;{\times PLI}_{m}}$$ (4)

Where CF_i_ is the pollution coefficient of heavy metal i; C_i_ is the measured value of heavy metal i; C_oi_ is the evaluation standard of heavy metal i, with the background values of soil elements in Inner Mongolia used in this study [37]; PLI is the pollution load index for a specific point; n is the number of heavy metals being evaluated; PLI_area_ is the pollution load index for a certain area; m is the number of sampling points in the contaminated area.

#### 2.4.3. Human health risk assessment.

To assess the health risks of exposure to heavy metals in soil through inhalation(ADIinh), dermal contact(ADIder), and Oral ingestion (ADIing), the model proposed by the U. S. Environmental Protection Agency [21,22] was used. The model is divided into non-carcinogenic and carcinogenic categories and evaluates both children and adults. with the exposure equations shown in (5), (6), and (7).


$${\text{ADI}}_{\text{ing}}=\frac{C\text{i}\times I\text{ng}R\times EF\times ED\times CF}{AT\times BW}$$ (5)

$${\text{ADI}}_{\text{inh}}\text{=}\frac{C\text{i}\times \;I\text{nh}R\times EF\times ED\times CF}{AT\times BW\times PET}$$ (6)

$${\text{ADI}}_{\text{der}}\text{=}\frac{C\text{i}\times \;SA\times SL\times EF\times ED\times ABS\times CF}{AT\times BW}$$ (7)

Where: Ci is the concentration of heavy metal i in the soil sample; IngR is the daily intake rate of soil (mg·d^−1^); EF is the exposure frequency (d·a^−1^); ED is the duration of exposure (a); CF is the conversion factor (kg·mg^−1^); AT is the average exposure time (d); BW is the average body weight (kg); InhR is the inhalation rate (mg·d^−1^); PET is the particulate emission factor (m^3^/kg); SA is the skin exposure area (cm^2^); SL is the skin adhesion factor (mg·cm^−2^); ABS is the skin absorption efficiency factor (unitless). The detailed exposure factors are shown in Table S1 in S1 File [2,38].

Non-carcinogenic effects are represented by the non-carcinogenic risk index (HQi) for individual heavy metal i and the total non-carcinogenic risk index (HI). Carcinogenic effects are represented by the carcinogenic risk index (CRi) for individual heavy metal i and the total carcinogenic risk index (CRI). The calculation formulas are as follows (8)(9)(10)(11):


$$\text{HQij }=\sum_{j=\text{1}}^{\text{3}}\frac{{\text{ADI}}_{ij}}{{\text{RfD}}_{ij}}$$ (8)

$$\text{HI}=\sum_{i=\text{1}}^{n}{\text{HQ}}_{i}$$ (9)

$$\text{CRi}=\sum_{j=\text{1}}^{\text{3}}{AD\text{I}}_{ij}\;\cdot {\text{SF}}_{ij}$$ (10)

$$\text{CRI}=\sum_{i=\text{1}}^{n}{\text{CR}}_{i}$$ (11)

Where: i represents an individual heavy metal; j represents an exposure pathway; RfD is the reference dose, mg·(d·kg)^-1^; SF is the slope factor, kg·d·mg-1; the specific reference values are provided in Table S2 in S1 File [31,39,40]HI > 1 indicates the occurrence of adverse non-carcinogenic risk effects, while HI < 1 indicates the absence of non-carcinogenic risk effects. The carcinogenic risk levels are classified as follows: Very low (< 10^−6^), Low (10^−6^ ~ 10^−5^), Medium (10^−5^ ~ 10^−4^), High (10^−4^ ~ 10^−3^), and very high (> 10^−3^) [31].

### 2. 5. Source appointment

#### 2.5.1. Principal component analysis（PCA）.

Principal component analysis (PCA), as a linear dimensionality reduction technique, can eliminate the multicollinearity between multiple heavy metal elements, thereby achieving dimensionality reduction and data information concentration [27]. The principal component calculation formula is shown in equation (12).


$$Z_{ij}=\sum_{k=\text{1}}^{p}a_{ik}\times c_{kj}$$ (12)

Where: aik represents the loading factor of source component k on element i; c_kj_ is the factor score of sample j on source component k. Once the results are obtained, rotation is performed until a reasonable outcome is achieved. The study employs orthogonal transformation to process the original loadings, making the interpretation clearer and more reliable.

#### 2.5.2. Positive matrix factorization model（PMF）.

The positive-definite matrix factorization model is a receptor model recommended by the U. S. Environmental Protection Agency for pollutant source apportionment [28]. The model operates based on matrix factorization and uses the least squares method to minimize the objective function Q. The model decomposes the o × i dimensional matrix (X_oi_) into a contribution matrix (G_ok_) and a factor matrix (F_ki_), as shown in equation (13) [31]:


$$X_{oi}\;=\sum_{k=\text{1}}^{p}G_{ok}\times {\;F}_{ki}+E_{oi}$$ (13)

Where: X_oi_ represents the concentration of the i-th element in the o-th sample; o and i refer to the number of samples and species, respectively. The matrix G_ok_ indicates the contribution of source k to sample o, while the matrix F_ki_ represents the concentration of the i-th element from source k. k is the number of factors, and E_oi_ is the residual matrix. The objective function Q is given by equation (14):


$$\text{Q}=\sum_{o=\text{1}}^{n}\sum_{i=\text{1}}^{m}\;\left(\frac{E_{oi}}{U_{oi}}\right)$$ (14)

In the equation, U_oi_ represents the uncertainty of species in each sample, with calculation formulas (15) and (16):


$$U_{oi}=\frac{\text{5}}{\text{6}}\times MDL_{c}\;\leq MDL$$ (15)

$$U_{oi}=\sqrt{{(\delta \;\times \;\text{ c})}^{\text{2}}+(\text{0}.\;\text{5}\;\times \text{ MDL}{)}^{\text{2}}}c>MDL$$ (16)

Where: MDL is the detection limit, δ is the relative standard deviation, and c is the element concentration.

## 3. Results and discussion

### 3.1. Description of soil properties and trace metals characteristics

Descriptive statistics for the A rongqi urban-agricultural transition zone are presented in Table S3 in S1 File. Soil pH ranged from 6. 65–7. 11, with an average of 6. 77. The Urbanizing Zone had the lowest pH at 6. 53, followed by Peri-urban Arable Land at 6. 64, and Urban at 6. 80. Compared to the 6. 36 pH in the black soil region of China [41], the soil pH differences were not significant, with a pH variation coefficient < 0. 1, indicating low soil acidification in the A rongqi urban-agricultural transition zone. Soil pH is influenced by factors such as parent material and land use, with fertilizer application being a major cause of soil acidification in China [3]. The lowest pH in the Urbanizing Zone and the value of 6. 80 in Urban indicate that urbanization causes much less acidification than agricultural land, suggesting that urbanization has not exacerbated the acidification of previously cultivated soils.

As shown in Table S3 in S1 File, soil SOM content ranged from 9. 18–49. 24 g/kg, with an average of 24. 23 g/kg. Urban had the lowest SOM value at 21. 54, followed by Urbanizing Zone at 25. 42, and Peri-urban Arable Land at 26. 82. Compared to the organic matter content in the black soil region of China from the second soil survey [42], SOM in the A rongqi urban-agricultural transition zone is significantly lower, indicating severe degradation. This is consistent with previous research findings [41]. In arable land, plant replenishment and tillage increase microbial abundance in soil aggregates, which to some extent promotes organic matter enrichment [43]. Peri-urban Arable Land has higher SOM than Urbanizing Zone, suggesting that urbanization has disrupted the original SOM input pathways, thus reducing SOM content.

As shown in Table S3 in S1 File, the average concentrations of Cd, Cu, Pb, Zn, As, Hg, Ni, Cr, Sb, Ba, Co, Mo, Sb, Sr, V, Tl, Zn, and Ag were 0. 11, 13. 25, 25. 96, 59. 48, 7. 72, 0. 02, 16. 20, 27. 54, 0. 62, 937. 90, 12. 68, 1. 69, 0. 62, 388. 70, 75. 16, 0. 65, 59. 48, 0. 11, 6. 65, and 2. 50 mg/kg, respectively. Cu, Hg, Ni, Cr, Sb, and Tl concentrations were lower than the provincial soil background values. Elements such as As, Ni, Cr, Co, Mo, Sr, and V exhibited high coefficients of variation, reflecting local hotspots or areas of enrichment for these elements. The average concentrations of Zn and Hg were higher than those in arable land in the black soil region of China [44], while the average concentrations of Cd, Cu, Pb, Zn, As, Hg, and Cr were significantly lower than those in industrial cities in the black soil region of China [45]. Cu, Co, As, Ni, Cr, and V were significantly higher than those in the black soil of post-mining restoration areas in Oklahoma [5]. With the exception of Mo, the average concentrations of other elements were higher than those in the Jebba Area of Fast-rising town’s mining restoration zone [22]This indicates that urbanization has led to the accumulation of various heavy metals in the black soil, but the pollution is significantly lower than that caused by mining development, intensive farming, and other pathways.

After the subdivision of the A rongqi urban-agricultural transition zone, the average concentrations of Cd, Hg, Ba, Mo, Tl, and Ag were highest in the Urbanizing Zone, followed by Peri-urban Arable Land and Urban. Cd concentrations exceeded the background values by two times in the Urbanizing Zone, Peri-urban Arable Land, and Urban. The average concentrations of Cu, Pb, As, Sb, and Sr showed the opposite trend to those of Cd and other elements. This is consistent with the conclusion that Cd is the primary pollutant in agricultural areas [46]. The variation in Cu, Cu, As, Zn, As, and Hg concentrations in the Urbanizing Zone was significantly greater than that in Peri-urban Arable Land and Urban, indicating that urbanization has led to the accumulation of these elements. However, the accumulation of external pollutants [47] has exacerbated the original pollution [48], and further evaluation of heavy metal contamination is needed.

### 3.2. Evaluation of trace metal contamination

**Fig 2:**
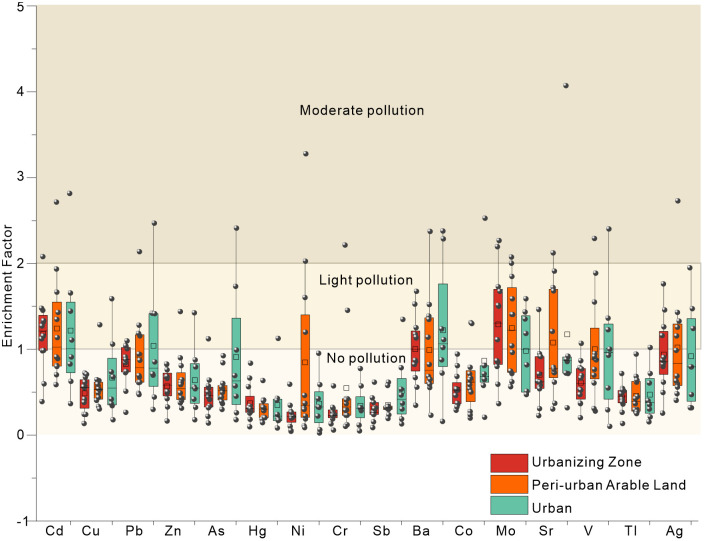
Enrichment Factor Evaluation of Subzones in the Urban-Agricultural Transition Zone.

The results of the Enrichment Factor (EF) and Pollution Load Index (PLI) are shown in Table S4 in S1 File and Fig 2. EF(Cd), EF(Ba), and EF(Mo) all reached moderate pollution levels, with Cd being the most prominent and requiring attention. In their provincial-scale heavy metal assessment in China identified Cd as the most common pollutant [48]. The subdivision within the transition zone indicated that Urbanizing Zone (Cd)> Peri-urban Arable Land (Cd)> Urban (Cd). Cd accumulation in Peri-urban Arable Land was significantly higher than in Urban, and the low variation of Cd in Peri-urban Arable Land (as shown in Table S3 in S1 File) suggests regional influences. The study suggests that Cd accumulation is widespread in black soil farmland, primarily due to agricultural activities [42,44]. This perspective was validated in the current study. light Cd pollution in the Urbanizing Zone (Table S4 in S1 File) and the variation in Table S3 in S1 File indicate that urbanization has exacerbated the original Cd pollution in farmland. Furthermore, although the urban-agricultural transition zone shows no pollution for Pb, Sr, V, Ag, etc., the subdivision within the zones reveals light pollution of Cd, Ba, and Mo in the Urbanizing Zone. light pollution of Cd, Mo, Sr, V, and Ag in Peri-urban Arable Land, and light pollution of Cd, Pb, Ba, and Sr in Urban were observed. Notably, light Pb pollution in Urban and Ag in Peri-urban Arable Land exhibited low coefficients of variation, suggesting that the accumulation was due to overall changes within the zones. The pollution point location percentage indicates that 8. 3% of Cd in the Urbanizing Zone presents moderate pollution risk. Peri-urban Arable Land has moderate pollution points for Cd, Pb, Ni, Cr, Ba, Mo, Sr, and Ag. Urban has moderate pollution risk points for Cd, Pb, As, Ba, Co, Sr, and V.

In Table S4 in S1 File, the Pollution Load Index (PLI) for the area is categorized as Moderate pollution (PLI = 1. 03), with Cd, Pb, Ba, Mo, and Ag being the main contributing elements. PLI for Peri-urban Arable Land is Moderate pollution (1. 22), and PLI for Urban is also Moderate pollution (1. 09). It is worth noting that the Urbanizing Zone (0. 95) indicates no pollution, which is inconsistent with previous evaluation results. Previous evaluations based on land use zoning suggested that urban expansion generally results in greater heavy metal accumulation than no pollution [49–51]. Current evaluations based on concentration enrichment of land use fail to account for the pollution accumulation before land use changes and the comprehensive effects brought by these changes. However, the classification of urban-agricultural transition zones allows for a more precise assessment of heavy metal accumulation, enhancing the sensitivity of pollution evaluation. The Urbanizing Zone is transformed from farmland, while Peri-urban Arable Land continues to undergo agricultural activities, which places its soil at higher pollution risk. Therefore, the classification of the Urbanizing Zone before and after land use changes is crucial for accurate pollution evaluation.

### 3.3. Human health risk assessment

As shown in Table S5 in S1 File, the non-carcinogenic risk exposure pathways indicate that for adults, the exposure pathway follows HQing > HQder > HQinh, while for children, it follows HQing > HQinh > HQder. Oral ingestion is the primary non-carcinogenic risk exposure pathway for both adults and children. This is consistent with the exposure pathways of heavy metals in urban areas, where children’s risks are higher than those for adults [52,53]. This may be related to children’s pica behavior and their higher breathing rates [54]. The average HI values for the 16 heavy metals in the Urban-agricultural transition Zone, as well as in each of the zones within the Urban-rural transition belt, are all less than 1, indicating that the effect of heavy metals in the black soil of the Urban-rural transition belt does not pose a non-carcinogenic risk to human health.

Due to the lack of available slope factors, only the carcinogenic risks of Cd, Pb, As, Ni, and Cr can be calculated. As shown in S6 and S7 in S1 File, in terms of carcinogenic risk, the heavy metals in the Urban farming transition zone present a medium carcinogenic risk for both children and adults, with children (7. 69E-05) facing a significantly higher risk than adults (1. 86E-05), with oral ingestion and dermal contact being the primary exposure pathways. Among the carcinogenic risk indices for different heavy metals, Cr ranks highest, followed by As. The CR values for Cr for adults (1. 06E-05) and children (6. 21E-05) are classified as medium, while the CR value for As in children (1. 44E-05) is also classified as medium, indicating that Cr and As are the primary carcinogenic risk elements for children, with Cr also being a major carcinogenic risk element for adults.

As shown in S6 and S7 in S1 File, under the regional divisions within the zone, the carcinogenic risk levels of As and Cr for adults and children differ significantly. The overall carcinogenic risk for adults: Peri-urban Arable Land＞Urban＞Urbanizing Zone. Peri-urban Arable Land presents medium risk from Cr and low risk from As for adults, Urban presents medium risk from As and risk from Cr for adults, and Urbanizing Zone presents low carcinogenic risk from both As and Cr for adults. The overall carcinogenic risk for children: Urban＞Urbanizing Zone＞Peri-urban Arable Land, with all three regions presenting medium carcinogenic risk from both As and Cr for children. Oral ingestion and dermal contact are the main exposure pathways in both Urbanizing Zone and Urban, while oral ingestion is the primary exposure pathway in Peri-urban Arable Land. As is a highly toxic element, and ingestion above a certain concentration can damage the brain and nervous system [55]. Excessive intake of Cr can also harm the respiratory and digestive systems. It is important to note that children have a poor ability to excrete toxins, resulting in greater harm [56]. Therefore, it should receive more attention. The carcinogenic risks from oral ingestion and dermal contact exposure pathways can be controlled by reducing the frequency and duration of contact with Urban soils.

### 3.4. Source identification and apportionment of trace metals in black soil

#### 3.4.1. Correlation analysis of soil heavy metals.

**Fig 3:**
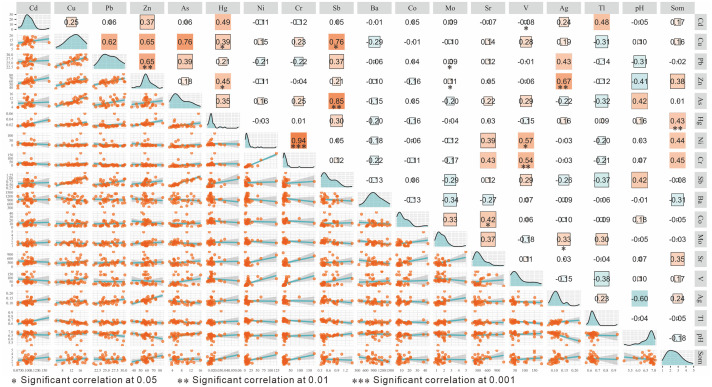
Plot of bivariate correlation of trace metals in urban farming transition zone.

Elements in the soil with the same source and behavior are generally significantly correlated with each other [46]. The correlation analysis results of elements in the urban-agricultural transition zone are shown in Fig 3. Ni is strongly positively correlated with Cr, and Zn is strongly positively correlated with Cu (P < 0. 001, R > 0. 5), indicating a high likelihood of having the same source. Zn is significantly positively correlated with Ag, Cu with Pb and Sb, As with Sb, and V with Ni and Cr (P < 0. 05, R > 0. 5), suggesting they may originate from the same source. The correlation relationships between V and As, Cu, Sb, Mo and Tl, Ag, S, Pb and As, Ag, Sb, Cd and Tl, Hg, SOM and Sr, Cr, Ni, Zn, and As and pH, Hg are not significant, with correlation coefficients R ranging from 0. 25–0. 49 (P > 0. 05), indicating the possibility of similar sources. Therefore, further analysis of heavy metal sources is needed.

#### 3.4.2. Source apportionment by the PCA and PMF model.

The KMO and Bartlett’s sphericity test results for the 11 elements meeting the testing criteria were examined (KMO = 0. 563, Sig = 0. 000 < 0. 001), ensuring suitability for principal component analysis. Principal component analysis identified five factors with eigenvalues greater than 1 (PC1 = 3. 90; PC2 = 3. 10; PC3 = 2. 18; PC4 = 1. 70; PC5 = 1. 44), which together explained 85. 23% of the total variance. The data after Varimax rotation are shown in Table S8 in S1 File. The PC1 explains 33. 26% of the variance, with loadings for elements Sb, As, and Cu being 0. 948, 0. 929, and 0. 807, respectively. The PC2 accounts for 23. 51% of the variance, with loadings for Zn, Ag, and Pb being 0. 889, 0. 865, and 0. 726, respectively. The PC3 explains 15. 74% of the variance, with Cr and Ni showing significant positive loadings of 0. 976 and 0. 973, respectively. Finally, the PC4 accounts for 12. 74% of the variance, with loadings for Cd, Hg, and Tl being 0. 90, 0. 672, and 0. 663, respectively.

**Fig 4:**
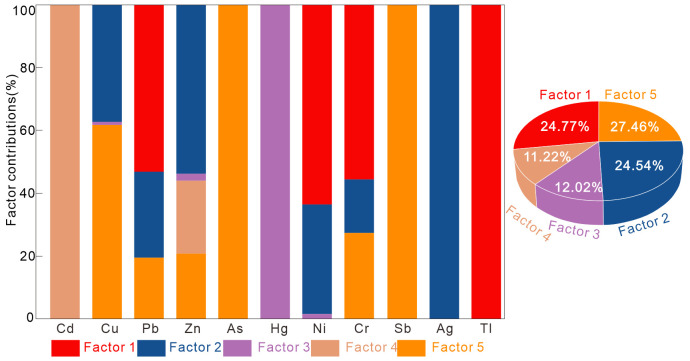
PMF model analysis of the factor distribution of trace metal elements and the contribution percentage of individual sources.

Source allocation analysis of 11 elements in soil was performed using PMF, ensuring that the signal- to-noise ratio of the selected elements exceeded 1, with four factors and residuals ranging from −3–3. The model yielded the best analytical performance (r² ranging from 0. 64–0. 98), as shown in Fig 4.

Factor 1 accounts for 24. 77% of the total pollution, with Tl, Ni, Cr, and Pb having the highest loadings, at 100%, 63. 6%, 55. 7%, and 53. 1%, respectively. Factor 2 accounts for 24. 54% of the total pollution source, with Ag and Zn having the highest loadings, at 100% and 53. 5%, respectively. Factor 3 accounts for 12. 02% of the total pollution source, with Hg being the main loading element, accounting for 100%. Factor 4 accounts for 11. 22% of the total pollution source, with Cd being the main loading element, accounting for 100%. Factor 5 accounts for 27. 46% of the total pollution source, with Sb, As, and Cu being the main loading elements, accounting for 100%, 100%, and 61. 7%, respectively.

The source profiles and contributions of the PCA and PMF models are shown in the figure. As shown in Table S8 in S1 File and Fig 4. The overlap in source contributions is relatively high, with Sb, Cu, and As contributing significantly in both PC1 and Factor 5. Sb, Cu, As, and Tl are negatively correlated, which is typically associated with anthropogenic sources [57]. Sb, As, and Cu in both PC1 and Factor 5 are highly representative, further confirming the consistency of the sources. The anthropogenic sources of Sb and As are largely attributed to the combustion of fossil fuels and traffic pollution [58,59]. Cu is often generated with the burning of fuel and brake pad wear [60], and the high concentration accumulation of these three elements in Urban, as shown in Table S3 in S1 File, also supports this view. Therefore, PC1 and Factor 5 are interpreted as traffic-related sources.

Ag and Zn have strong representativeness in both PC2 and Factor2, Ag and Zn are significantly correlated, while the correlation between Ag and Pb is not significant, suggesting that Pb may have other potential sources. Studies have shown that the use of phosphate fertilizers can lead to widespread Pb accumulation [61], Chemical fertilizers and pesticides are major contributors to Zn pollution [62], The widespread use of organophosphorous pesticides in agricultural production can lead to Ag heavy metal contamination [63]. Combining the high concentration accumulation of these three elements in Peri-urban Arable Land, PC2 and Factor2 are interpreted as originating from agricultural sources.

There is a correlation between Cd, Hg, and Tl in PC4, but it is not significant, indicating a potential common source. Tl and Hg show a weak correlation, suggesting the presence of other sources. The moderate variation of Hg and the low variation of Cd and Tl also suggest a trend of different sources. The distinction between Factor1 and Factor3, as well as Factor4, further validates this view. Cd and Tl exhibit areal accumulation, while Hg is a point source of pollution, This study suggests that Cd pollution from agrochemicals and pesticides is distributed areally, whereas sources such as electroluminescent source manufacturing and urban solid waste are point sources of pollution [64,65]. Tl pollution primarily originates from parent material [57,66], It can also accumulate due to excavation and landfilling [66]. The combustion of fossil fuels, the electronics industry, and household waste are all likely to cause the accumulation of Hg [51], In Northeast China, Hg typically originates from coal combustion, exhibiting a strong organic affinity, and is deposited into the soil or binds with clay minerals via atmospheric deposition [67]. Combined with the high concentration accumulation of these three elements in the Urbanizing Zone, Factor3 is interpreted as an urban combustion source, and Factor4 is interpreted as an agricultural source prior to urbanization. PC4 is explained as a mixed source from urban construction and inheritance.

Ni and Cr exhibit a significant correlation (r = 0. 94). The study suggests that Cr and Ni originate from natural weathering processes and are significantly influenced by the parent material weathering process. The combination of the two is typically referred to as natural element markers [10]. Based on this, PC3 and Factor1 are interpreted as natural sources.

There are five sources of heavy metals in the soils of the urban-agricultural transition zone, with the following contribution rankings: transportation sources (27. 46%), parent material sources (24. 77%), agricultural sources (24. 54%), urban combustion sources (11. 22%), and legacy agriculture sources (11. 22%).

## 4. Conclusion

This study provides a comprehensive assessment of ecological and health risks associated with trace metals in black soils under urbanization pressure. Key findings, which offer critical insights for targeted environmental management, are synthesized as follows:

Urbanization has altered soil properties and metal burdens, but not uniformly. While it did not exacerbate acidification, it led to a decline in soil organic matter. The process has intensified pre-existing cadmium (Cd) pollution in agricultural land and caused the accumulation of multiple heavy metals. However, the overall contamination level remains less severe than that typical of industrialized cities.

Pollution and health risks are driven by distinct priority elements and show spatial specificity. Ecological risk is primarily attributed to Cd, Pb, Ba, Mo, and Ag, with a general pattern of mild pollution. Notably, the contributing elements vary across land-use types (e.g., Cd-Ba-Mo in Urbanizing Zones; Cd-Mo-Sr-V-Ag in Peri-urban Arable Land). For human health, chromium (Cr) and arsenic (As) are identified as the key carcinogenic drivers, with the highest risks to adults in Peri-urban Arable Land and to children in Urban areas. This clearly establishes a priority order for element-specific control.

The dominant pollution sources are traffic, agricultural activities, and parent material, as consistently indicated by receptor models (PCA and PMF). The significant role of agricultural sources, even in the evolving Urbanizing Zone, highlights the legacy effect of past land use.

## Supporting information

S1 File Supporting Tables S1–S8 provided in a single Word file. (ZIP)
